# Draft Genome Sequence of *Gordonia sihwensis* Strain 9, a Branched Alkane-Degrading Bacterium

**DOI:** 10.1128/genomeA.00622-16

**Published:** 2016-06-23

**Authors:** Lisa M. Brown, Thusitha S. Gunasekera, Richard C. Striebich, Oscar N. Ruiz

**Affiliations:** a University of Dayton Research Institute, Dayton, Ohio, USA; b Air Force Research Laboratory, Aerospace Systems Directorate, Fuels and Energy Branch, Wright-Patterson AFB, Ohio, USA

## Abstract

*Gordonia sihwensis* strain 9 is a Gram-positive bacterium capable of efficient aerobic degradation of branched and normal alkanes. The draft genome of *G. sihwensis* S9 is 4.16 Mb in size, with 3,686 coding sequences and 68.1% G+C content. Alkane monooxygenase and P-450 cytochrome genes required for alkane degradation are predicted in *G. sihwensis* S9.

## GENOME ANNOUNCEMENT

*Gordonia sihwensis* strain 9, a Gram-positive actinomycete bacterium, was isolated from raw sludge from a wastewater treatment facility in Dayton, OH, USA after enrichment with hydrocarbons. This strain was shown to metabolize a gamut of normal and branched alkanes. The type strain of *G. sihwensis* was originally isolated from a wastewater-treatment bioreactor in South Korea (1). Based on BLAST analysis (http://blast.ncbi.nlm.nih.gov/Blast.cgi) of the 16S rRNA gene sequence, *G. sihwensis* S9 is 100% similar to *G. sihwensis* NBRC 108236, DSM 44576, and SPR2, 99% similar to *G. neofelifaecis* NRRL B-59395, and 98% similar to *G. alkanivorans* NBRC 16433. The gyrase subunit A gene (*gyrA*) of strain S9 is 100% similar to *G. sihwensis* NBRC 108236 but only 92% and 84% similar to *G. neofelifaecis* and *G. alkanivorans*, respectively. *Gordonia* species, including *G. alkanivorans* have been shown to actively degrade branched and aromatic hydrocarbons and contain desulfurization pathways (2–4). Due to the high capacity for hydrocarbon degradation and possible use in biodesulfurization applications, we have sequenced the genome of *G. sihwensis*, to increase the understanding of this versatile bacterium.

*G. sihwensis* S9 was sequenced on a Roche 454-GS Junior platform using a whole-genome shotgun (WGS) approach, producing 360,174 reads. The Roche *de novo* assembly software aligned reads into 348 large (>500 bp) contigs with an average depth of 40 reads and *N*_50_ of 25,140 bp. The largest contig extended 183,625 bp. The draft genome sequence was 4,155,610 bases in length with a G+C content of 68.1%. The NCBI Prokaryotic Genome Annotation Pipeline (http://www.ncbi.nlm.nih.gov/genome/annotation_prok/) predicted 4,115 genes, including 3,686 coding sequences (CDSs), 51 tRNAs, and 372 pseudogenes. BLAST analysis indicated three copies of the 16S rRNA gene. Rapid genome annotations using the RAST server (5) assigned the coding sequences to 383 subsystems, of which amino acids and derivatives (*n* = 403 CDSs); carbohydrates (*n* = 308); cofactors, vitamins, prosthetic groups, and pigments (*n* = 243); protein metabolism (*n* = 231); fatty acids, lipids, and isoprenoids (*n* = 229); RNA metabolism (*n* = 108); nucleosides and nucleotides (*n* = 88); and virulence, disease, and defense (*n* = 80) were most abundant.

The NCBI Prokaryotic Genome Annotation Pipeline predicted 3 alkane 1-monooxygenase genes (*alkB1*). Immediately downstream of *alkB1* in contig 00033, 2 rubredoxin genes and a rubredoxin reductase gene were identified. Similar three-part genetic system for the degradation of normal and branched alkanes has been observed in *P. aeruginosa* and *Rhodococcus* sp., respectively (6, 7). Eight P-450 genes with three showing at least 68% homology to *Alcanivorax borkumensis* P-450 alkane hydroxylase cytochromes were predicted. *A. borkumensis* cytochromes are involved in the degradation of branched alkanes (4). Other hydrocarbon degradation genes observed included benzene 1,2-dioxygenase, catechol 1,2-dioxygenase, phenol 2-monooxygenase, 2-nitropropane dioxygenase, 4-hydroxybenzoate 3-monooxygenase, and multiple mono and dioxygenases. Also, the alkanesulfonate monooxygenase and dimethyl sulfone monooxygenase genes involved in biodesulfurization were detected. This genome will facilitate the study of mechanisms by which *Gordonia* species adapt to and degrade hydrocarbons.

### Nucleotide sequence accession number.

This project has been deposited at DDBJ/EMBL/GenBank under the accession no. JZDP00000000.
